# Comparative efficacy and safety of JAK inhibitors and leflunomide in rheumatoid arthritis: A protocol for systematic review and meta-analysis

**DOI:** 10.1097/MD.0000000000032154

**Published:** 2022-12-09

**Authors:** Xiaogang Zhang, Mingming Zhang, Zhiqiang Wang, Yanqing Liu, Xing Feng, Liu Yang, Yajing Wang, Juan Liu, Dongbao Zhao

**Affiliations:** a Department of Rheumatology and Immunology, Changhai Hospital, the Second Military Medical University, Shanghai, China; b Department of Rheumatology and Immunology, the 980th Hospital of PLA Joint Logistics Support Force, Shijiazhuang, China.

**Keywords:** JAK inhibitors, leflunomide, meta-analysis, rheumatoid arthritis

## Abstract

Rheumatoid arthritis is the most prevalent systemic autoimmune disease among the rheumatic inflammatory musculoskeletal diseases. Currently, there is no reliable evidence regarding the comparisons of anti-rheumatic effects between leflunomide and Janus kinase inhibitors. We performed a protocol for systematic review and meta-analysis to compare the efficacy and safety of Janus kinase inhibitors and leflunomide in rheumatoid arthritis.

**Methods:**

This review has been reported following the preferred reporting items for systematic reviews and meta-analyses protocol. We will search 7 electronic databases to identify relevant studies from inception to November, 2022, which includes PubMed, MEDLINE, Embase, Cochrane Clinical Trials Database, Web of Science, China National Knowledge Infrastructure, and Chinese Biomedical Literature Database. Risk of bias will be assessed according to the Cochrane Risk of Bias Tool. The Grading of Recommendations Assessment, Development and Evaluation system will be used to judge the overall quality of evidence supporting outcomes in this work. Data analysis was performed using Reviewer Manager 5.4.

**Results:**

The results of this systematic review will be published in a peer-reviewed journal.

**Conclusion:**

This systematic review will provide evidence to judge whether Janus kinase inhibitors is superior to leflunomide in patients with in rheumatoid arthritis.

## 1. Introduction

Rheumatoid arthritis (RA) is an autoimmune disease mainly characterized by arthritis and accompanied by multiple systemic damage. In addition to joint symptoms, it can also lead to vasculitis, subcutaneous nodules, blood system abnormalities, cardiopulmonary dysfunction, or other tissues and organs involved. ^[1–3]^ Heredity, environment, and immune disorders are major factors in the development of the disease. Due to its complexity, its pathophysiological mechanism has not yet been fully elucidated, and a good management of chronic diseases for RA requires multidisciplinary methods.^[1,4]^ So far, there is still no completely effective treatment for RA. In recent years, a lot of progress has been made in the development and utilization of disease control drugs for RA, especially biological targeted therapeutic drugs, this has significantly improved the clinical symptoms and disease activity of RA patients.^[5–6]^

Leflunomide (LEF) is one of the most commonly used disease-modifying anti-rheumatic drugs (DMARDs) for the treatment of RA in clinical practice,^[7–8]^ and it was listed as one of the top three recommended treatments for rheumatoid arthritis by the American College of Rheumatology in 2008[9]. Because its early use can significantly improve the prognosis of patients with RA, LEF has been effectively used for early RA and has been shown to significantly inhibit disease as assessed progression by joint imaging.^[10]^

Janus kinase (JAK) inhibitor, the latest class of drugs in the treatment of RA, is a small molecule biological drug, which has been widely used in clinical practice. It is the first oral targeted synthetic biological agent for RA treatment.^[11]^ JAK inhibitors can selectively inhibit JAK kinase and block JAK/STAT pathway, thereby inhibiting signal transduction of various inflammatory cytokines. JAK inhibitors have been accepted by many patients because of their more "smart" mechanism of action and oral route of administration.^[12]^ Currently, there is no reliable evidence regarding the comparisons of anti-rheumatic effects between leflunomide and JAK inhibitors. We performed a protocol for systematic review and meta-analysis to compare the efficacy and safety of leflunomide and JAK inhibitors in RA.

## 2. Methods

### 2.1. Study registration

This systematic review has been registered in PROSPERO (CRD42022372872), which will be conducted in accordance with preferred reporting items for systematic review and meta-analysis protocols 2015 statement.^[13]^ Given that the meta-analysis is a secondary research which based on some previously published data, ethical approval is not necessary for our research.

### 2.2. Inclusion criteria for study selection2.2.1 types of study

To compare the efficacy and safety of leflunomide and JAK inhibitors in RA, only related randomized controlled trials (RCTs) will be included in the evaluation. Others such as case reports, animal experiments, non-RCTs, or RCT protocol will be excluded.

#### 2.2..1. Types of participants.

The patients range are limited to adult patients diagnosed with RA, regardless of age, gender, educational status or racial restrictions.

#### 2.2..2. Types of intervention.

Intervention groups receive JAK inhibitors and control groups receive leflunomide. There is no restrictions with respect to dosage, frequency, duration, or follow-up time of treatment.

#### 2.2..3. Types of outcome measures.

The primary outcomes are visual analogue scale, modified health assessment questionnaire score, erythrocyte sedimentation rate and C-reactive protein levels. The secondary outcomes are liver-function tests, routine urine tests, renal-function tests and adverse events.

### 2.3. Search strategy

We will search 7 electronic databases to identify relevant studies from inception to November, 2022, which includes PubMed, MEDLINE, Embase, Cochrane Clinical Trials Database, Web of Science, China National Knowledge Infrastructure, and Chinese Biomedical Literature Database. Meanwhile, we will also search Chinese Clinical Trial Registry and ClinicalTrials.gov for ongoing trials with unpublished data. The search strategy in PubMed is shown in Table 1.

**Table 1:** Search strategy (PubMed).

#1 rheumatoid arthritis [Title/Abstract]
#2 arthrorheumatism [Title/Abstract]
#3 rheumarthrosis [Title/Abstract]
#4 rheumatoidarthritis [Title/Abstract]
#5 RA [Title/Abstract]
#6 #1 OR #2 OR #3 OR #4 OR #5
#7 leflunomide [Title/Abstract]
#8 Arava [Title/Abstract]
#9 #7 OR#8
#10 Janus kinase inhibitors [Title/Abstract]
#11 JAK inhibitors [Title/Abstract]
#12 Ruxolitinib [Title/Abstract]
#13 Tofacitinib [Title/Abstract]
#14 Oclacitinib [Title/Abstract]
#15 Baricitinib [Title/Abstract]
#16 #10 OR #11 OR #12 OR #13 OR #14 OR #15
#17 #6 AND #9 OR #16

### 2.4. Study selection

**Figure 1.**
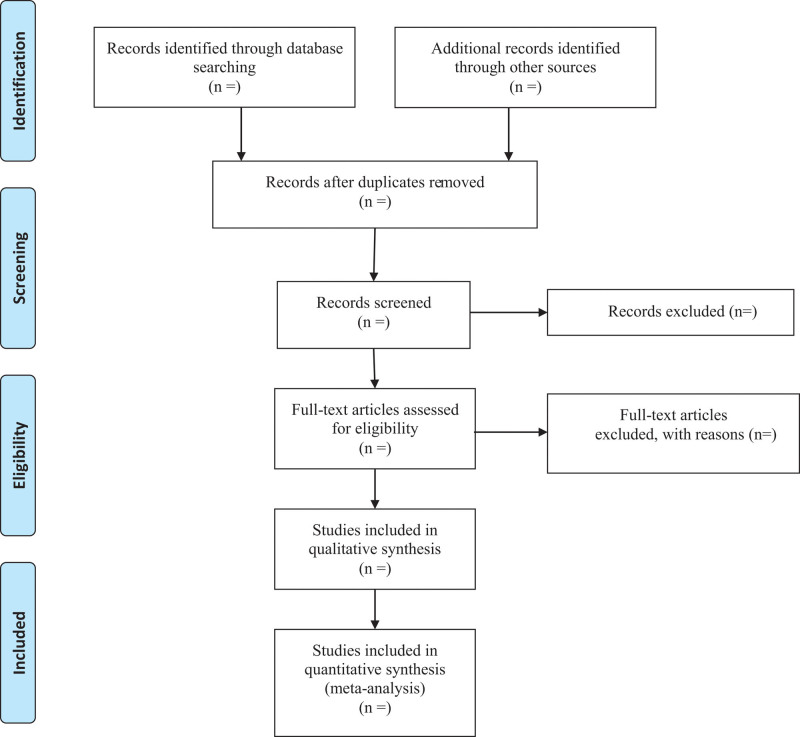
Flow diagram.

We will export the identified records in databases into EndNote X9 software and use this to identify duplicates. After removing duplicates, the retrieved records will be checked independently by 2 reviewers, who will apply the eligibility criteria based on the title and abstract. Where a study is potentially eligible, the full text will be obtained and checked independently by 2 reviewers to identify the eligible studies. Any disagreements will be discussed and resolved in discussion with a third reviewer. Details of the selection procedure for the studies are shown in the PRISMA flow chart (Fig. 1).

### 2.5. Data extraction and management

Two reviewers will take charge of data extraction and management according to the retrieval strategy, including the title of the study, the journal, the year of publication, the name of the first author, general information, study design, intervention, outcomes, and adverse events. If there is any disagreement between 2 reviewers in the data extraction, the group will arbitrate and make decisions together.

### 2.6. Dealing with missing data

When it comes to missing or unclear data, we will try our best to contact the corresponding author for more detailed information. If it fails, we will analyze it based on available data.

### 2.7. Risk of bias assessment

Two authors will independently assess the risk of bias of the included studies based on the bias risk assessment tool recommended in the Cochrane “Risk of bias” assessment tool.^[14]^ Including 7 items: random sequence generation, allocation concealment, blind participants and personnel, blind assessment of results, incomplete result data, selective reports, and other biases. The results in each field will be divided into 3 levels: low bias risk, high bias risk, and unclear bias risk.

### 2.8. Assessment of quality of evidence

The Grading of Recommendations Assessment, Development and Evaluation system will be used to judge the overall quality of evidence supporting outcomes in this work.^[15]^ And the quality of evidence will be defined as high, moderate, low, or very low.

### 2.9. Statistical analysis

Two researchers respectively entered the data into the Reviewer Manager 5.4 software. Mean differences with a 95% confidence interval were calculated to assess the effect size for continuous outcome data. Risk ratio with a 95% confidence interval were used as effect size for dichotomous data. Inverse variance method and Mantel-Haenszel analysis method were used for continuous variables and dichotomous variables, respectively. The heterogeneity among the trials was assessed for significance with Q and quantified with I^2^. Statistically significant was set at the *P* value < 0.10. If the studies were homogeneous or the statistical heterogeneity was low, we used the fixed effect-model. While, random-effects model was applied when the statistical heterogeneity was moderate or high. If the heterogeneity of the included studies is large, subgroup analyses will be performed on the basis of different interventions, controls, durations of treatment, and outcome measures.

## 3. Discussion

Rheumatoid arthritis is one of the most common autoimmune diseases in clinic.^[16,17]^ At present, there is still a lack of specific and effective treatment methods, and patients often need to take medicine for a long time. However, with the progress of chronic disease management model and the emergence of more effective therapeutic drugs, especially the continuous development and utilization of more and more biological agents, the treatment cost is getting lower and lower, and ultimately more patients benefit.^[18–19]^

## Author contributions

**Conceptualization:** Mingming Zhang.

**Data curation:** Zhiqiang Wang.

**Formal analysis:** Yanqing Liu.

**Investigation:** Yanqing Liu.

**Methodology:** Xing Feng.

**Software:** Liu Yang.

**Supervision:** Yajing Wang.

**Visualization:** Juan Liu.

**writing – original draft:** Xiaogang Zhang.

**Writing – review & editing:** Dongbao Zhao.
